# Rotational Preference in Gymnastics

**DOI:** 10.2478/v10078-012-0042-4

**Published:** 2012-07-04

**Authors:** Thomas Heinen, Damian Jeraj, Pia M. Vinken, Konstantinos Velentzas

**Affiliations:** 1 German Sport University Cologne, Institute of Psychology Am Sportpark Müngersdorf, Germany.

**Keywords:** Unterberger-Fukuda test, laterality, non-experts, near-experts, experts

## Abstract

In gymnastics, most skills incorporate rotations about one or more body axes. At present, the question remains open if factors such as lateral preference and/or vestibulo-spinal asymmetry are related to gymnast’s rotational preference. Therefore, we sought to explore relationships in gymnast’s rotation direction between different gymnastic skills. Furthermore, we sought to explore relationships between rotational preference, lateral preference, and vestibulo-spinal asymmetry. In the experiment n = 30 non-experts, n = 30 near-experts and n = 30 experts completed a rotational preference questionnaire, a lateral preference inventory, and the Unterberger-Fukuda Stepping Test. The results revealed, that near-experts and experts more often rotate rightward in the straight jump with a full turn when rotating leftward in the round-off and vice versa. The same relationship was found for experts when relating the rotation preference in the handstand with a full turn to the rotation preference in the straight jump with a full turn. Lateral preference was positively related to rotational preference in non-expert gymnasts, and vestibulo-spinal asymmetry was positively related to rotational preference in experts. We suggest, that gymnasts should explore their individual rotational preference by systematically practicing different skills with a different rotation direction, bearing in mind that a clearly developed structure in rotational preference between different skills may be appropriate to develop more complex skills in gymnastics.

## Introduction

In gymnastics, most skills incorporate rotations about one or more body axes (Arkaev and Suchilin, 2004). These skills range from simple leaps with turns to more complex acrobatic maneuvers and artistic flight elements with rotations about their body axes. Gymnasts decide at a young age whether to rotate leftward or rightward about their longitudinal axis. This preference is usually maintained during their career (Arkaev and Suchilin, 2004; Sands, 2000). Nevertheless, the question remains open if factors such as lateral preference and/or vestibulo-spinal asymmetry are related to gymnast’s rotational preference (Arkaev and Suchilin, 2004; Heinen et al., 2010). Therefore, the purpose of this study was twofold. First, we sought to explore relationships in gymnast’s rotational preference between gymnastic skills, which are developed early in the gymnast’s career and are therefore important for the career development. Second, we sought to explore relationships between rotational preference, lateral preference and vestibulo-spinal asymmetry in gymnastics.

There are no rules in gymnastics that constrain the rotation direction about the longitudinal axis in one direction, because each element that contains a rotation about the longitudinal axis can in principal be performed in both directions (Arkaev and Suchilin, 2004). However, in skills that will be developed later in a gymnast’s career such as the Kasamatsu on vault, it is required to rotate about the longitudinal and the transverse axis in a well-defined manner. If a gymnast is for instance not able to rotate leftward in a round-off and rightward in a forward twisting somersault, or vice versa, he or she will not be able to perform a Kasamatsu on vault.

Sands (2000) observed female gymnast’s rotational preference on different skills during national competitions in the USA. The study revealed no tendency for either a leftward or a rightward rotational preference. However, one main result was that the rotation direction in twisting somersaults was consistent across elements with a similar structure. The author furthermore observed that about 80% of athletes, who rotated rightward in round-off, rotated leftward in a twisting backward somersault, and vice versa. Heinen et al. (2010) found a similar pattern of results. These results may at least in part be explained by perceptual similarity (Arkaev and Suchilin, 2004; Heinen et al., 2010). When the vestibular system is placed upside down like in a handstand, the information from the vestibular system is inverted. One feels rotating leftwards but is instead rotating rightwards (Heinen et al., 2010). However, this phenomenon is generally accepted in gymnastics coaching, since the athlete should try to maintain his or her (subjective) rotational preference in order to prevent orientation problems and blackout phenomena (Day and Thatcher, 2006; Sands, 2000). Therefore, it is likely to assume that most gymnasts develop a rotational preference in skills performed in an upright posture, such as a straight jump with a full turn which is contrary to their rotational preference in skills which are performed mainly upside down, such as a handstand with a full turn. However, Sands (2000) did not assess rotational preference in other gymnastic skills, like a straight jump with a full turn, which is one of the first learned gymnastic skills, and thus may possibly be important for choosing a leftward or rightward direction of rotation about the longitudinal axis. Additionally, the author did not assess other parameters like measures of laterality or vestibulo-spinal asymmetry that may be related to rotational preference. It can be suggested that lateral preference may be related to rotational preference (Martin and Porac, 2007; Starosta, 2000), because learners in general choose movement strategies in new tasks in favor of their lateral preference (Serrien et al., 2006).

There is comprehensive research done on the relationship between rotational preference and lateral preference in general. In an investigation by Scharine and McBeath (2002), participants had to travel through a “T-maze”, searching for a hidden object at the end of the maze on either the right or the left side. Volunteers did not know that the hidden object was on both sides. The chosen direction was recorded and the experiment was stopped as soon as the participant had chosen one direction. Additionally, participants were assessed on different measures of laterality. The results revealed, that 73% of the participants chose the right side for searching the hidden object, and handedness was the best predictor of participants’ choice. However, the study sample was unequally distributed on handedness, making it hard to draw general conclusions on the relationship between handedness and rotational preference. In another study, right-handed women showed a slight tendency for a rightward turning preference and left-handed person showed a leftward turning preference. However, the effects were not statistically significant (Bradshaw and Bradshaw, 1988).

In a longitudinal study, Golomer et al. (2009) found that the majority of a sample of prepubertal girls showed a leftward rotational preference, while there were no significant relationships to measures of laterality. In a group of classical dance students, only one dancer showed a consistent leftward rotational preference, whereas the remaining dancers showed a rightward rotational preference. The authors concluded that this pattern of results is likely due to the training history and the choice of the supporting leg because in general, dance students are asked to perform pirouettes to the right (Laws and Sugano, 2008). In another investigation by Brown et al. (1983), participants’ (51 gymnasts and 120 non-athletes) rotational preference was assessed in four simple gymnastic movements. Measures of laterality such as handedness and eye dominance were also assessed. The authors observed a small relation between lateral preference and a jump turn and an additional small relationship between hand dominance and rotational preference on a swivel-hip maneuver on the trampoline in the athlete sample. In a recent study by Heinen et al. (2010), it was found that eye dominance and foot dominance may account for differences in rotational preference in a sample of intermediate gymnasts. Nevertheless the questions remain if such results generalize to gymnasts of different age and expertise levels, or if other factors may also account for differences in athletes’ rotational preference.

One additional factor that may account for rotational preference could be the asymmetry of the vestibulo-spinal system. For instance, when people are asked to step repeatedly without moving, with eyes closed, they tend to side deviate, rotate about their longitudinal axis, and move forward (Nyabenda et al., 2004). The most common explanations for this deviation are seen in differences in lateral preference and/or an asymmetry of the vestibular system on either the central or the peripheral level (Toussaint et al., 2008). However, authors in general find no significant or only weak correlations between participant’s deviation when stepping and lateral preference (Toussaint et al., 2008). Nevertheless, an asymmetry of the vestibular system could on the one hand (if there is no pathological change in the vestibular system) potentially increase people’s sensitivity for rotating in one direction or another. On the other hand, it could be the result of an adaptation of the vestibular system if a person was exposed to a high amount of rotations about the longitudinal axis. From this point of view, we decided to assess vestibulo-spinal asymmetry as an additional potential factor related to participant’s rotational preference. If this asymmetry is related to rotational preference (either as a cause for rotational preference or as a result of adaptation processes), gymnasts should more often rotate in their preferred rotation direction when stepping on the spot.

Taken together, there is no clear evidence concerning relationships between rotational preference and other factors such as lateral preference or vestibulo-spinal asymmetry (Heinen et al., 2010). However, there is evidence on relationships in rotational preference between skills with different demands (Sands, 2000). From this point of view, it was first hypothesized that gymnasts maintain their (subjective) rotational preference in different gymnastic elements. Second, it was assumed that lateral preference is not related to rotational preference in gymnasts (Brown et al., 1983). Our third assumption was that vestibulo-spinal asymmetry is positively related to rotational preference in gymnasts. Since one may speculate that differences in rotational preference and differences in relationships between rotational preference and factors such as lateral preference or vestibulo-spinal asymmetry can or cannot be moderated by a skill level, it was decided to test all hypotheses in gymnasts on different skill levels (Arkaev and Suchilin, 2004; Golomer et al., 2009).

## Material and Methods

### Participants

A total of 90 gymnasts participated in our study (n = 30 non-experts, n = 30 near-experts, n = 30 experts). The number of subjects was derived from a power analysis (Cohen, 1988), when expecting a medium effect with a Type I error probability of 5% and a Type II error probability of 20%.

Non-experts, near-experts and experts differed in the skill level but not in age (mean age = 16 ± 4 years) or years of training (11 ± 6 years). Non-experts were able to execute rather basic gymnastic exercises such as a round-off, a somersault or a handspring. They participated in regional competitions. However, they were not able to perform more complex elements such as a twisting somersault or release-regrasp exercises. Near-experts were able to execute advanced gymnastic exercises, such as twisting somersaults or basic release-regrasp exercises with a rotation about one body axis. They participated in regional and national competitions. However, none of them was able to perform twisting somersaults with more than two twists or double somersaults with one or more twists about the longitudinal axis. Experts were former and active members of the extended German National Gymnastics Team. They participated in national and international competitions. They were able to perform challenging elements, such as twisting somersaults with more than two twists about the somersault axis, double somersaults with one or two twists about the somersault axis, and release-regrasp exercises with a rotation about one or two body axes.

We decided to recruit the participants of all three groups in a way that there was an equal distribution in rotational preference in straight jump with a full turn on the floor (n = 15 left, and n = 15 right for each group), because this skill is one of the first skills learned in a gymnast’s career that incorporates a rotation about the longitudinal axis (German Gymnastics Federation, 2008). All gymnasts were informed about the purpose and the procedures of the study and gave their written informed consent prior to the study. The study was carried out according to the ethical guidelines and with the approval of the ethical committee of the German Sport University Cologne.

### Material and Measures

Rotational Preference Questionnaire. All gymnasts in our study were asked to complete a questionnaire on their rotational preference. We were interested in gymnast’s rotational preference in three different gymnastic elements: (1) straight jump with a full turn, (2) round-off, and (3) handstand with a full turn. It was decided to select these three skills, because first, they are among the first skills a gymnast learns already in the beginning of his/her career. Second, since we were interested in differences in rotational preference about the longitudinal axis in different gymnastic skills, one skill should incorporate an isolated rotation about the longitudinal axis in upright stance (straight jump with a full turn), whereas another skill should incorporate an isolated rotation about the longitudinal axis when being upside down (handstand with a full turn). In addition, we selected the round-off, because this element incorporates a rotation about the longitudinal axis when being upside down, but the exercise begins in an upright position. If gymnasts develop a rotational preference with regard to a “subjective” maintenance of rotation direction in different skills due to perceptual similarity, they should show a leftward rotation in upright stance and a rightward rotation in the handstand and vice versa.

**Figure 1:**
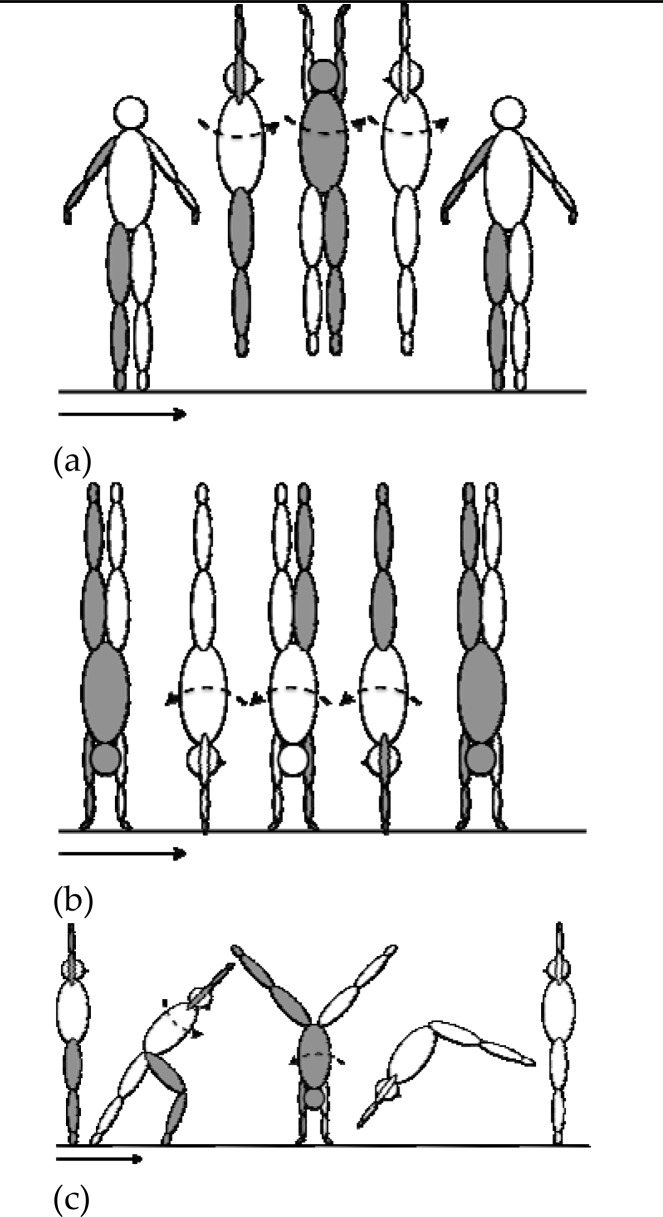
Illustration of a leftward rotation in the three assessed gymnastic skills. The right leg, the right arm, the back, and the back of the head are marked in grey. (a) Straight jump with a full turn. (b) Handstand with a full turn. (c) Round-off.

In the questionnaire, the definition of rotation direction in the three gymnastic elements was always relative to the gymnasts’ longitudinal axis (Figure 1). Rotation was defined as “left”, when the body front (the waist) was moved to this side where the left body side (the left shoulder) was at the beginning of the rotation. Rotation was defined as “right”, when the body front (the waist) was moved to this side where the right body side (the right shoulder) was at the beginning of the rotation.

Figure 1 indicates the rotation direction in the three aforementioned gymnastic exercises. Participants were asked to indicate their preferred direction of rotation as either “left” or “right” for the straight jump with a full turn, and the handstand with a full turn. Regarding the “roundoff”, which is a cartwheel with a half turn, so that the athlete looks to this side from where he starts performing the element, and landing on both feet at the same time, gymnasts were asked to indicate which hand they place first on the ground when performing the element.

This was done because gymnasts are used to imitate the hand placement in the round-off rather than indicating their preferred twist direction. The direction of rotation in the roundoff results from a direct function of the first hand touching the ground in this skill. If the gymnast places his or her left hand at first on the ground the resulting direction of rotation will be right and vice versa. During the completion of the questionnaire, participants were allowed to imitate the movement if needed.

Lateral Preference Inventory. To evaluate lateral preference we used a German version of the Lateral Preference Inventory (Coren, 1993). This questionnaire assesses lateral preference in four dimensions: (1) eyedness, (2) earedness, (3) handedness, and (4) footedness. Gymnasts were asked to respond to 16 questions related to the aforementioned dimensions, indicating their corresponding lateral preference (“left” or “right”; Büsch et al., 2009). Four items are used to assess each dimension. An example for a question related to the dimension of handedness is: “Which hand do you use for drawing?” In summary, a score ranging between −4 (left consistent type) to +4 (right consistent type) can be calculated for each dimension. In order to classify each gymnast as either left or right consistent, we calculated the sum score of the questionnaire in each individual case by summing up the scores of all four dimensions. The distribution of all sum scores was subjected to a median split procedure (median = 10) from which each gymnast could be classified as either left consistent or right consistent.

Unterberger-Fukuda Stepping Test. The stepping test is a clinical test to examine vestibulo-spinal asymmetry (Fukuda, 1957). We used a modified version of the Unterberger-Fukuda Stepping Test. When performing this test, the gymnast was asked to step 100 times on the spot without moving in any direction. During the test, the gymnast wore earplugs and a sleep mask to eliminate visual and auditory feedback. To control tactile feedback, the test was performed on an even and homogeneous ground. After 100 steps, the gymnast was asked to rest in place. We assessed the rotation direction (“left” or “right”) of the gymnast in each individual case. Since all gymnasts of our sample rotated either to the left or to the right (Nyabenda et al., 2004), the rotation direction could be unambiguously determined in each individual case.

### Procedure

The research was conducted in three phases. In the first phase, the gymnasts arrived at the lab. Then they completed and signed the informed consent form. The second phase was the data acquisition. It took place in a separate room without environmental influences. Each gymnast was examined individually. The room was equipped with a table and a chair, and there was enough space for participants to simulate gymnastic movements and to perform the Unterberger-Fukuda Stepping Test. The gymnast completed the Rotation Preference Questionnaire, the Lateral Preference Inventory and the Unterberger-Fukuda Stepping Test. The sequence of tests was randomized for each gymnast in order to prevent possible order effects. The complete duration of the data acquisition took about 20 minutes. The gymnast was allowed to rest at will. In the third phase and after completion of the tests, the gymnast was debriefed.

### Data analysis

We defined a significance criterion at the level of p < .05. In order to examine relationships in rotational preference between the three assessed gymnastic elements, we conducted separate frequency analyses by calculating the Pearson contingency coefficient (C) for the three groups (Blaikie, 2003). The same procedure was done to examine relationships between rotational preference in the straight jump with a full turn, the lateral preference and the rotation in the Unterberger-Fukuda Stepping Test. Through the calculation of Holm’s correction, the inflation of Type I and Type II errors was controlled (Ludbrook, 1998).

## Results

### Relationships in rotational preference between gymnastic skills

First, we assumed that gymnasts would maintain their (subjective) rotational preference in all assessed gymnastic elements. In order to examine relationships in rotational preference between the several gymnastic exercises, separate frequency analyses by calculating the Pearson contingency coefficient for the three groups were conducted.

**Figure 2:**
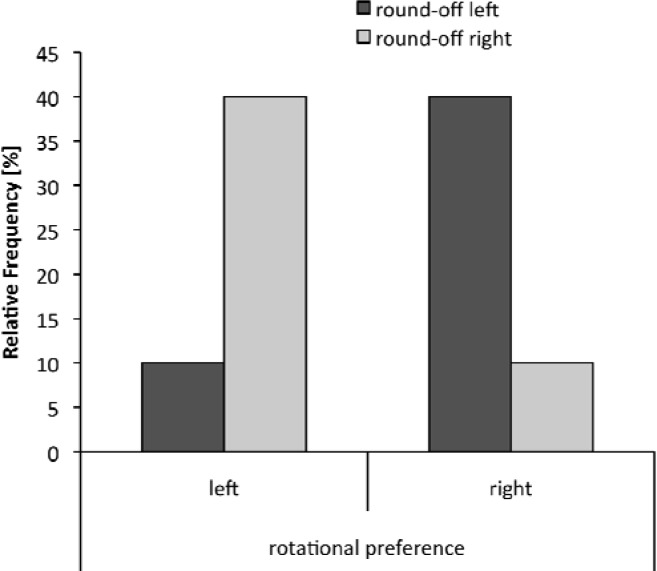
Frequency distribution between rotational preference in the straight jump with a full turn and the round-off in near-experts.

The analysis revealed neither a significant relationship (Pearson contingency coefficient - C) for rotational preference between the straight jump with a full turn and the round-off, C = 0.258, p = .14, nor for rotational preference between the straight jump with a full turn and the handstand with a full turn in non-experts, C = 0.135, p = .45. With regard to the near-expert group, we found a significant relationship for the rotational preference between the straight jump with a full turn and the round-off, C = 0.520, p < .01, but only a small, yet not significant, tendency for rotational preference between the straight jump with a full turn and the handstand with a full turn, C = 0.262, p = .13. Experts exhibited significant relationships in rotational preference between the straight jump with a full turn and the round-off, C = 0.570, p < .01, as well as for rotational preference between the straight jump with a full turn and the handstand with a full turn, C = 0.470, p < .01. Near-experts and experts who rotate to the left in the straight jump with a full turn, more often rotate to the right in the round-off and vice versa. Experts who rotate to the left in the straight jump with a full turn also rotate more often to the right in the handstand with a full turn and vice versa (Figure 2).

### Relationships between rotational preference and lateral preference

Our second assumption was that lateral preference is not related to rotational preference. In order to examine relationships between rotational preference (in terms of rotation direction in the straight jump with a full turn) and lateral preference, separate frequency analyses were conducted by calculating the Pearson contingency coefficient for the three groups.

**Figure 3:**
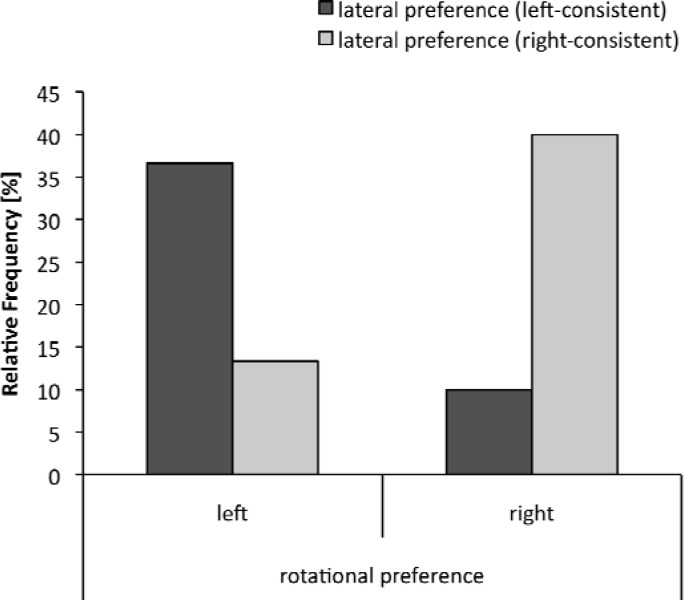
Frequency distribution between rotational preference in the straight jump with a full turn and the lateral preference in non-experts

The analysis revealed neither a significant relationship for near-experts, C = 0.132, p = .46, nor for experts, C = 0.670, p = .72. Near-expert and expert gymnasts who were either left or right consistent did not rotate more often to the left or right in the straight jump with a full turn. However, the analysis revealed a significant relationship for non-experts, C = 0.47, p < .01. Non-experts who were left-dominant in lateral preference more often rotated leftward in the straight jump with a full turn and vice versa (Figure 3).

### Relationships between rotational preference and vestibulo-spinal asymmetry

Our third assumption was that vestibulo-spinal asymmetry is positively related to rotational preference in gymnasts. Separate frequency analyses were carried out by calculating the Pearson contingency coefficient for the three groups, in order to examine relationships between rotational preference (in terms of rotation direction in the straight jump with a full turn) and vestibulo-spinal asymmetry.

**Figure 4:**
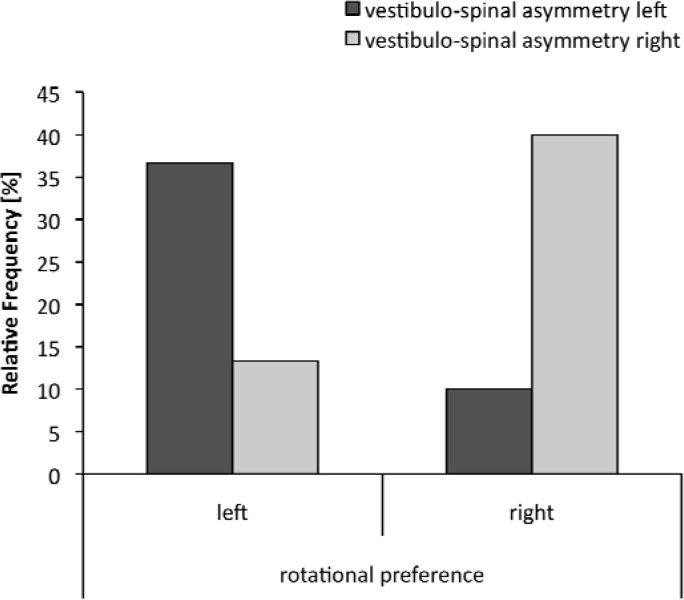
Frequency distribution between rotational preference in the straight jump with a full turn and gymnasts rotation in the Unterberger-Fukuda Stepping Test as a measure of vestibulo-spinal asymmetry

The analysis revealed neither a significant relationship for non-experts, C = 0.149, p = .41, nor for near-experts, C = 0.067, p = .71. However, a significant relationship was found for experts, C =0.470, p < .01, indicating, that expert gymnasts who rotate to the left in the straight jump with a full turn, more often rotate to the left in the Unterberger-Fukuda Stepping Test and vice versa (Figure 4).

## Discussion

There were two main aims of this study. On the one hand, we sought to explore relationships in gymnast’s rotational direction in different gymnastic elements, which are developed early in a gymnast’s career. On the other hand, we aimed at examining relationships between rotational preference and two plausible influencing factors, namely lateral preference and vestibulo-spinal asymmetry. We hypothesized that gymnasts maintain their (subjective) rotational preference in all assessed gymnastic elements, and that there is no significant relationship between lateral preference and rotational preference. In addition, we assumed that vestibulo-spinal asymmetry is positively related to rotational preference in gymnasts. Non-experts, near-experts and experts completed a rotational preference questionnaire, a lateral preference inventory, and the Unterberger-Fukuda Stepping Test. The results revealed that near-experts and experts more often rotated rightward in the straight jump with a full turn when rotating leftward in the round-off and vice versa. The same relationship was found for experts when relating the rotational preference in the handstand to the rotation preference in the straight jump with a full turn. Lateral preference was not related to rotational preference in the near-expert and expert group. However, lateral preference was positively related to rotational preference in non-expert gymnasts, indicating that non-experts who were left-consistent more often showed a leftward rotational preference in the straight jump with a full turn and vice versa. Vestibulo-spinal asymmetry was positively related to rotational preference in experts, indicating that experts who rotated to the right in the straight jump with a full turn, more often showed a rightward rotation in the Unterberger-Fukuda Stepping Test.

In addition to the results of Sands (2000), expert gymnasts exhibit a clear pattern of rotational preference in different gymnastic elements. In the authors’ opinion this may first and foremost be explained by perceptual similarity, but not by laterality. Perceptual similarity may occur when a gymnast rotates in different directions about his or her longitudinal axis either in upright stance or when being upside down, so that the vestibular information is similar in both cases (Heinen et al., 2010; von Laßberg et al., 2003). This may explain why we found a relationship in rotation direction between the straight jump with a full turn, the handstand with a full turn, and the round-off in experts. The same relationship was found in rotational preference between the round-off and the straight jump with a full turn in near-experts. It could be the case that this is a result from a coaching strategy aiming at teaching athletes to maintain their rotational preference throughout several skills in order to prevent orientation problems and blackout phenomena (Day and Thatcher, 2006), as well as in order to prepare the development of more complex skills such as the Kasamatsu on vault. However, we did not find a significant relationship in rotational preference between the three assessed skills in non-experts. Taken the different results in the three groups together, one may speculate that a preferred rotation direction is developed during gymnasts’ learning process and may strongly be related to the demands of the training environment (Golomer et al., 2009).

According to the results of the lateral preference inventory and the rotational preference, our results for experts and near-experts are similar to the findings of Brown et al. (1983) as well as to the findings of Golomer et al. (2009) who found no clear relationships between lateral preference and rotational preference in skilled athletes. However, we found a significant relationship between lateral preference and rotational preference in non-experts. Especially in gymnastics, athletes define their rotation direction about the longitudinal axis and maintain this preference throughout their career (Arkaev and Suchilin, 2004; Sands, 2000). One important factor for this decision could be lateral preference (Martin and Porac, 2007), because learners in general choose movement strategies in new tasks in favor of their lateral preference (Serrien et al., 2006). This could be true for non-experts who may not have the ultimate aim to learn more complex movements with a well-defined rotation about the longitudinal axis later in their career. However, this may not be the case for experts and near-experts, if the training environment enforces gymnasts to develop more complex skills later in their career, such as the Kasamatsu on vault, it is required that the gymnast rotates about the longitudinal axis in a well-defined manner. If a gymnast is for instance not able to rotate leftward in a round-off and rightward in a forward twisting somersault, or vice versa, he or she will not be able to perform a Kasamatsu on vault.

All gymnasts in our sample rotated either to the right or the left when performing the Unterberger-Fukuda Stepping Test, however, only in experts the rotation direction was significantly related to the rotational preference in the straight jump with a full turn. One may speculate that this pattern of results reflects more an adaptation process in experts, rather than a causal relationship between vestibulo-spinal asymmetry and rotational preference, since near-experts did not show a significant relationship between rotational preference and vestibulo-spinal asymmetry. Given that the experts in our sample were exposed to the largest training amount and therefore were better trained in performing rotations about the longitudinal axis in several skills, their vestibular system may have been adapted to the rotation training. It may of course also be possible that vestibulo-spinal asymmetry causes a rotational preference in several skills and therefore differentiates between gymnasts on different skill levels or age groups. This question cannot be answered by the data of our study, but it should be integrated in subsequent studies.

We are aware of several critical issues in our study and want to highlight two specific aspects. First, we assessed rotational preference, lateral preference, and vestibulo-spinal asymmetry. However, one may speculate about further factors, which may either influence rotational preference or are at least related to rotational preference, such as functioning of the vestibular system and the cerebellum (Taniewski, 1995) or the ability of mental rotation (Amorim et al., 2006). Subsequent studies should therefore incorporate different assessment methods in their designs. The ultimate goal could be to have a differentiated test battery to predict the rotational preference of each individual gymnast. This test battery should then be administered to male and female gymnasts on different skill levels, and in different age groups. Second, we recruited participants who had already developed their rotational preference. Another possibility could be to train persons for a longer period with regard to different rotation directions in different skills. Two groups of participants with an equal distribution on lateral preference or vestibulo-spinal asymmetry could be trained for a preferred rotation direction. It could be conceivable that gymnasts of two of these groups (e.g. right-handed with a left rotation preference and left-handed with a right rotation preference or vice versa) will learn skills with rotations about the longitudinal axis faster or execute these movements more precisely. This could help to explain the influence rather than the relationship of factors such as lateral preference or vestibulo-spinal asymmetry on rotational preference in gymnastics.

## Conclusion

We agree with the conclusion of Sands (2000) that a gymnast should have the opportunity to experiment and develop his or her rotational preference in order to prevent orientation problems. We state that this could be rather critical for complex gymnastic elements than for simple gymnastic movements (Arkaev and Suchilin, 2004), since first, from a biomechanical and psychological point of view, it seems to be functional to maintain rotational preference in a series of acrobatic elements (Prassas et al., 2006) with regard to perceptual similarity, and second, because more complex skills such as the Kasamatsu on vault are based on gymnastic movements an athlete learned already early in his or her career.
